# Effects of organic mercury on *Mytilus galloprovincialis* hemocyte function and morphology

**DOI:** 10.1007/s00360-020-01306-0

**Published:** 2020-09-26

**Authors:** Maria Giovanna Parisi, Jessica Pirrera, Claudia La Corte, Mariano Dara, Daniela Parrinello, Matteo Cammarata

**Affiliations:** Marine Immunobiology Laboratory, Department of Earth and Marine Sciences, University of Palermo, Viale delle Scienze, Edificio 16, 90128 Palermo, Italy

**Keywords:** Bivalve, Bioindicators, Hemocytes, Biomarkers, Phagocytosis, Toxic metals

## Abstract

**Abstract:**

Filter-feeding organisms accumulate xenobiotics and other substances in their tissues. They can be useful as sentinel organisms in biomonitoring of the marine compartment. Bivalve cellular immunity is ensured by phagocytosis and cytotoxic reactions carried out by hemocytes in a network with humoral responses. These can be affected by chemical contaminants in water that can be immunosuppressors also at a low concentration increasing the sensibility to pathogens. This work is an attempt to individuate cellular markers for pollution detection, investigating the effect of methylmercury (CH_3_HgCl) at different concentrations on the activity and hemocyte morphology of the Mediterranean mussel, *Mytilus galloprovincialis*. We assessed the effect of three sub-lethal concentrations of the organometal on the cellular morphology, the efficacy of phagocytosis toward yeast cells, the alteration of the lysosomal membrane and the ability to release cytotoxic molecules. The results provide information on the alteration of hemocyte viability, modification of the morphological and cytoskeletal features and besides the cellular spreading, intrinsic ability of motile cells was used as a complementary investigation method. Exposure to the contaminant affected the percentage of phagocytosis and the phagocytosis index. Moreover, morphological and cytoskeleton alteration, caused by the pollutant, leads to reduced ability to incorporate the target and adhere to the substrate and the low ability of cells to retain neutral red could depend on the effects of methylmercury on membrane permeability. These results reinforce the use of the Mediterranean mussel as model for the evaluation of environmental quality in aquatic ecosystems integrating the novel information about hemocyte functions and morphology sensibility to organic mercury.

**Graphic abstract:**

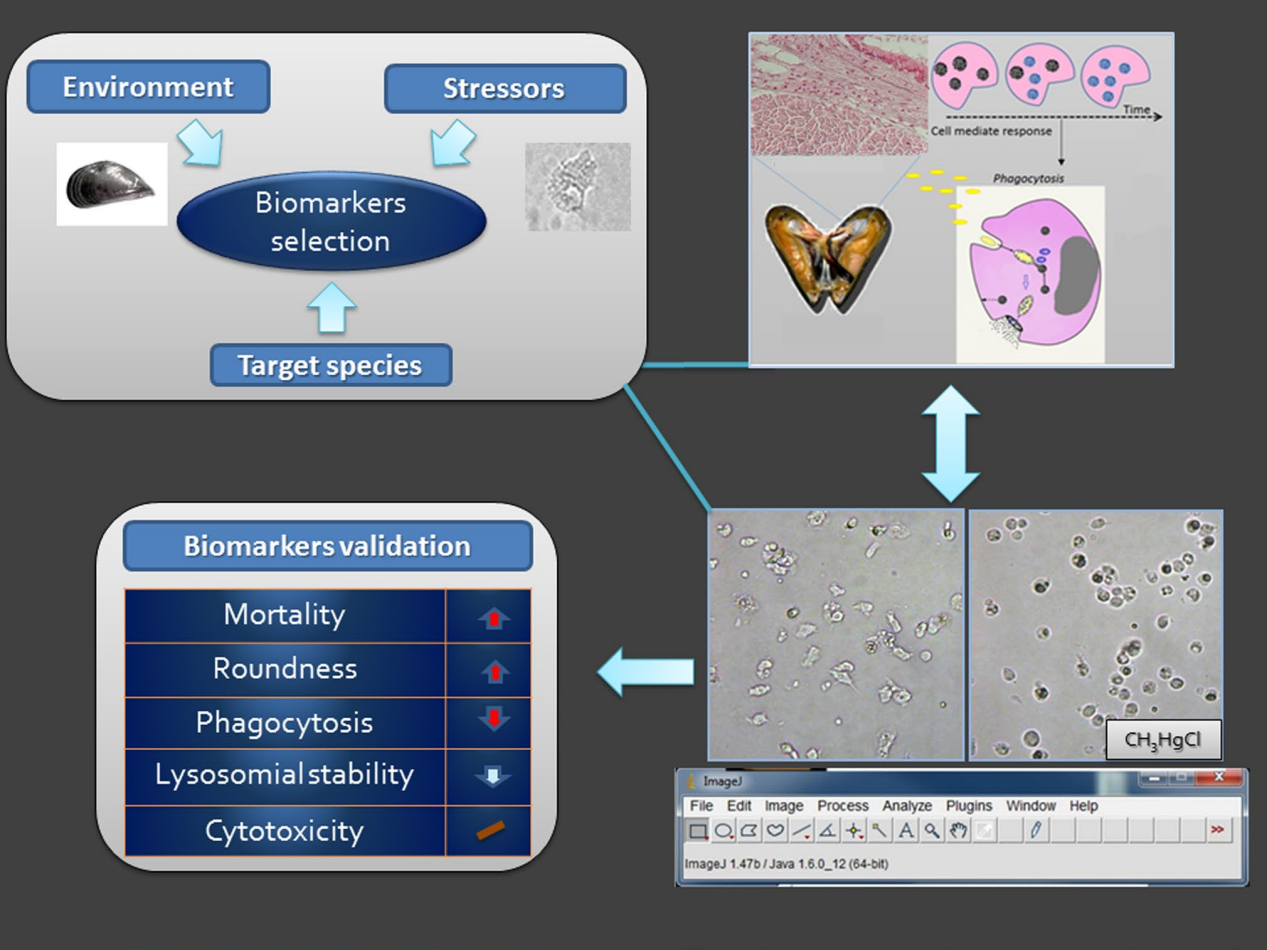

## Introduction

Human activities have caused the release of large quantities of metals in the environment, especially in the marine compartments (Jakimska et al. 2011; Richir 2016). The presence of these pollutants in marine water is due to the weathering of soils and rocks, mining, wastes, sludge residues, and oil burning (Singh et al. 2004; Guerra-García and García-Gómez 2005). As a result, with the increasing awareness of environmental issues, many questions have been raised on heavy metals—on their bioavailability and bioaccumulation and on their toxicity in the marine environment.

The contaminants and their accumulation or toxicity can be investigated in the environmental matrix and in biological tissues by the use of animal models validated as bio-monitoring tools of the ecosystem (Parmar et al. 2016). Additionally, the biochemical, cellular, and physiological responses (biomarkers) of the organisms (Hamza-Chaffai 2014) are valid and predictive instruments for estimating the exposition effect prior to alterations at the population and community levels (Walker et al. 2012; Khatri and Tyagi 2015). Biomarkers provide information on the molecular mechanism of toxicity and due to their measurable change in response to xenobiotics (at molecular, biochemical, cellular, physiological, pathological, or behavioural level), they are considered early indicators in biological systems (Allen and Moore 2004). They can highlight pre-pathologic alteration before other effects, disease, mortality or population changes (Connon et al. 2012), playing an important role in the definition, classification, and involvement or recovery strategies of contaminated sites.

Marine invertebrates are exposed to metals in marine water in both dissolved and particle phases. Xenobiotics can be adsorbed mostly in benthic filter feeding organisms directly through the body surfaces but also through the ingestion and digestion of food.

Bivalves are used as sentinel organisms because of their wide geographical distribution, ability to tolerate a range of environmental stressors and accumulate xenobiotics (Bresler et al. 1999; Fernández et al. 2012; Beyer et al. 2017; Caricato et al. 2019; Gornati et al. 2019), and they are largely used in field and laboratory analysis to individuate defensive, genotoxic, clastogenic, and even histopathological alterations as responses to augmented polluting stressors (Sánchez-Chardi et al. 2009; Cappello et al. 2019).

The Research Center for the evaluation of Good Environmental Status in the marine environment (JRC, 2010) required by the Marine Strategy Framework Directive (MSFD, 2008) recommends the use of the mussel as a sentinel in biomonitoring because of its “diagnostic and prognostic capacity on environmental quality”.

Oysters and mussels are species widely utilized in toxicological tests at different structural levels, from genes to individuals. Using them as bioindicators, several biomarkers have been validated like the larval-embryo developments, the growth, clearance and survival rates and changes in the density population (Viarengo et al. 2007; Klobučar et al. 2008).

In molluscs, metal accumulation causes lysosomal enlargement in the digestive cells, inducing mechanisms of metal detoxification like the increase of the synthesis of the cytosolic protein reduced glutathione (GSH) or metallothioneins (MTs).

Mercury exposition on filter feeder mollusks induce structural changes and permeability in cell membrane, alteration in a number of enzymatic reactions and imbalance in the levels of vital inorganic cations (Sivaramakrishna and Radhakrishnaiah 2000).

There are evidences of the effects of the inorganic form of mercury on growth, sexual maturation and reproductive success (Thain 1984). Brock (1992) reported Hg exposure effect on *Cerastoderma spp.* observing that the biosynthesis of porphyrin precursors, tS-amino levuleic acid (ALA) and porphobilinogen (PBG), perturbed in in vivo and in vitro experiments.

Additionally, mussels showed a higher capacity for mercury (Hg) accumulation compared to other bivalve’s species, such as oyster and clams (Briant et al. 2017).

Metals, coming from industrial wastewater discharged in rivers, may contain immuno-suppressors of marine organisms, enhancing autoimmune diseases; the effects depend on the dose, length, and time of exposure (Holladay and Smialowicz 2000; Dietert 2009; Winans et al. 2011).

Several environmental factors can influence defensive mechanisms and immunotoxicology is responsible for the understanding of how chemical, biological, physical and physiological factors alter the development of immune systems in humans and wildlife (Rhind 2009; Mohmand et al. 2015; Parisi et al. 2017).

Previous studies have individuated the potential immunological risk set by a range of chemicals of anthropogenic sources on vertebrates (Davis et al. 2002) and their toxicity can be relevant, for example, inorganic mercury causes renal lesions, neurotoxicity and cardiovascular disorders; moreover, its organic form (methylmercury) is the most toxic species inducing serious central nervous system dysfunctions (Briant et al. 2017).

A vast bibliography has treated the effects of these chemicals on birds and mammals, besides the domesticated species. However, not many investigations have been carried out on the effects of xenobiotics and their impact on invertebrate organisms, which constitute the largest number of species in the marine ecosystem (Landrum et al. 2001; Markich et al. 2002; Markman et al. 2007).

The toxicity of methylmercury (MeHg) could adversely impact the survival of the animals, lowering their resistance to stress. The hemocytes circulating in the hemolymph represent the early defensive lineage acting through phagocytosis, nodulation, encapsulation, cytotoxicity and hemolymph coagulation (Falleiros et al. 2003; Parisi and Cammarata 2014; Parrinello et al. 2016). About that, the viability, the physiology, the numbers and ratio of the different kinds of hemocytes are very important for homeostasis.

One of the first targets of organic mercury immunotoxicity in invertebrates is the hemocytes (Calisi et al. 2009). Hence, the cellular modifications of these hemocytes serve as biomarkers of polluted environmental conditions (Calisi et al. 2009; Hook et al. 2014).

Another effect that can derive from bivalve exposure to pollutants is the alteration of morpho-functional properties (Brousseau et al. 1999). Previous papers reported increased roundness of the immune cells with decreased number of pseudopods in hemocytes of animals (mussels and snails) exposed to mercury (Marchi et al. 2004; Leomanni et al. 2016). Also, sub-lethal methylmercury concentration affects rapidly the hemocytes of the Ascidian *Styela plicata*, suggesting an immunosuppression activity (Cammarata et al. 2007). These two variations, alteration in the number of cells and morphometric change, are considered instruments for revealing the sub-lethal stress conditions caused by organometals.

In a previous comparative study (Bellante et al. 2016) on Mediterranean filter feeder species and chemical elements in the water column, we showed the capability of a commercial bivalve *M. galloprovincialis* to accumulate a certain amount of the elements, including MeHg, as a consequence of the feeding behavior, reporting the bio-accumulation factor (Baf) calculated as the ratio of concentration of metal measured in the tissue of the organisms.

Here we want investigate the effect of exposure of sublethal concentration of CH_3_HgCl on hemocyte function and morphology of *M. galloprovincialis* especially in relation to the cell viability, and phagocytic and cytotoxic activities to validate the cellular response of the bivalve in our coastal areas as biomarker of exposure to organometal.

## Materials and methods

### Hemolymph collection

Mussels *M. galloprovincialis* (5–6 cm long) were kept in tanks with oxygenated water [dissolved oxygen (DO) 8 mg L^−1^; salinity 28‰; temperature 20 ± 2 °C]. The hemolymph was extracted (800 μl for each animal) from the posterior adductor muscle with a 1-ml sterilized syringe containing Alsever solution anticoagulant (200 μl; Na_3_C_6_H_5_O_7_, 27 mM, d-glucose 115 mM, NaCl 336 mM, EDTA 9 mM) and placed in plastic tubes.

Hemocyte count was carried out using the Neubauer chamber and adjusted to 1 × 10^6^ cells/ml for each sample.

After centrifugation (400×*g*; 10′; 4 °C), the hemocytes were resuspended in the same volume of marine solution (MS) (12 mM CaCl_2_; 11 mM KCl; 26 mM MgCl; 45 mM Tris; 38 mM HCl; 0.45 M NaCl, pH 7.4).

### Exposure of hemocytes to MeHg in vitro

Methylmercury master solution was prepared by dissolving the salt in MS at a concentration of 10^–3^ M.

Methylmercury working solutions were prepared by diluting the master solution with MS in the following concentrations: 10^–7^, 10^–6^, 10^–5^ and 10^–4^ M. The hemocytes (1 × 10^6^ cells/ml) were aliquoted in a 500 μl volume of MS and centrifuged at 400×*g* at 4 °C. The pellets were dissolved in working methylmercury marine solutions (MeMS 10^–7^ M, 10^–6^ M, 10^–5^,10^–4^ M). As control, a sample of same quantity of hemocytes was dissolved in MS.

### Viability and cell morphology

After exposition for 30 min, the hemocytes treated were placed on glass slides and their morphology observed under Nomarsky differential interference contrast microscopy (Diaplan, Leika, Wetzlar). The morphological alterations were observed by comparison to the shape of the hemocytes kept in MS (control).

The mortality of the hemocytes was evaluated by the Trypan blue test, and the cell physiology and the cell life cycle stage were revealed by the acridine orange/ethidium bromide (AO/EB) staining. The dead cells were determined by adding Trypan blue solution (0.01% in MS) to the medium. The hemocyte suspension (90 μl at 1*10^6^ cells/ml) was incubated with Trypan blue solution and observed by a light microscope at 40 × magnification each 5 min for 30 min.

To carry out the AO/EB staining, acridine orange and ethidium bromide solutions (l00 μg/ml) were mixed in the ratio 1:1. A volume of 25 μl of cell suspension was incubated with 1 μl of staining solution (AO/EB) and observed on a slide under a fluorescence microscope using a fluorescein filter at 40 × magnification. The apoptotic cells turned green with the chromatin condensate and nuclear fragmentation, orange with fragmented nuclei and necrotic cells. Orange color with nuclear morphology instead suggested the living cells without condensed chromatin. All observations were carried out in three biological replicates for each sample, each individual sample evaluation have been replicate three times (statistical replicates).

The roundness was calculated using “Image J”, an open-source software for digital image processing developed at the National Institute of Health (NIH). This software can calculate area and pixel value statistics of user-defined selections. The circularity parameter formula used was 4 × pi × (area/perimeter). A value equal to 1 indicates a perfect circle while values greater than 1 means elongated or non-circular shaped. Values tending to 0.0 indicate a polygon naturally elongated. Invalid values, due to the presence of small particles, were removed, excluding the particles with areas below a certain limit. The images obtained by the optical microscope were converted to jpeg 8-bit after they were digitalized by means of Image J. The evaluation was triplicated per each image.

### Phagocytosis fluorescence quenching in vitro assay

Phagocytosis assay was carried out, by adapting Parrinello et al. (2017), using the commonly known baker's yeast, *Saccharomyces cerevisiae* (Sigma), as a target. The yeast suspension (0.25%; w/v) was prepared in distilled water (1 × 107 cells ml^-1^), autoclaved for 15 min, washed twice (2000×*g*, 5 min, 4 °C), and incubated with eosin Y (4-bromo-fluorescein) for 1 h at 20 °C to a final concentration of 0.05%.

The yeast cells were washed four times and resuspended to a final concentration of 0.125% (w/v) in PBS and stored at 20 °C for a maximum of 2 weeks.

Yeast cells, hemocyte suspension in MS (100 μl at 1 × 10^6^ cells ml^−1^) and MeMS (10^–7^ M, 10^–6^ M, 10^–5^ M) were mixed (v/v) in 1-ml tube and incubated, gently shaking for 20 min at 20 °C.

To point out the phagocytosis, 50 μl of quenching solution (Trypan blue 2 mg ml^−1^, crystal violet 2 mg ml^−1^ in 0.02 citrate buffer, pH 4.4, containing NaCl 33 mg ml^−1^) was added to the cellular suspension. The cells were observed by Nomarski differential interference contrast optics and fluorescent apparatus (450–490-nm filter) (Diaplan, Leika, Wetzlar, D). The results were expressed as percentages of phagocytic cells containing yeasts and the phagocytic index as the mean number of yeasts incorporated by each phagocyte.

### Neutral red (NR) uptake assay and neutral red staining

The hemocyte suspension was prepared as previously reported (10^6^ cells/ml). A volume (200 μl) of cells suspended in MS and MeMS (10^–7^ M, 10^–6^ M, 10^−5^ M) was placed in a well of a flat-bottomed microplate (Nunc) in triplicate. The blank was prepared with 200 μl of acetic acid and ethanol; in another well, a negative control with 180 μl of acetic acid and ethanol was prepared.

Neutral red aliquots (C_15_H_17_C_l_N_4_, Sigma-Aldrich), 0.33% of 10 μl, were prepared by dissolving the salt in PBS containing NaCl (2%) and adding the mixture in each well. The plate was incubated (30 min, 10 °C).

After incubation, the plate was centrifuged (250×*g*; 5 min), the supernatant removed, and the plate washed twice with PBS buffer. After that, solutions of acetic (1%) and ethanol (50%) were added and the plate was incubated for 15 min at 20 °C in the dark. The absorbance was measured at 550 nm (RT-2100C Microplate Reader Rayto). The neutral red retention was expressed as ODT/mg^−1^ ml^−1^ protein referred to as the hemocyte content (Repetto et al. 2008).

The staining procedure with NR was carried out after in vitro exposition of the hemocytes to MeMS and MS.

A volume of 100 μl of each sample was placed on a slide and the debris-containing MS was discarded.

After cell adhesion, 60 μl of NR (Merck, Darmstadt, Germany) solution in MS (8 mg/ml) was added. The living hemocytes were directly observed; in fact, the dye stains the acid compartments of the living cells (Bancroft and Gamble 2002). After incubation (10 min), the slides were analyzed under light microscopy to observe the released dye from the lysosome and other alteration of these organelles in at least 50% of the observed granulocytes.

### Plaque-forming cell assay (PFC) and hemocyte cytotoxic assay (HCA)

Originally described for the human cell B-type (Cunningham and Szenberg 1968), the PFC has been adapted for the hemocytes of invertebrates (Cammarata et al. 1997). The test was performed as described: a volume of hemocyte suspension (50 μl; 1 × 10^6^ cells/ml) was mixed with a rabbit erythrocyte suspension (5%) in MS and the final suspension was rapidly observed in a chamber between two slides. The chamber was manufactured by placing three stripes of bioadhesive tapes on the border of a cover-slide and placing on it a slide; each chamber had an approximate volume of 100 µl. After incubation (15 min, 20 °C), the mixture was observed under a phase-contrast microscope. The control was an erythrocyte suspension (10 μl in 50 μl of MS).

The cytotoxic assay against red blood cells (RBC) as target was performed as described by Cammarata et al. (1997). A volume of 20 μl of hemocyte suspension in MS (effector cells: 1 × 10^6^ cells/ml) was mixed with a volume of freshly prepared erythrocyte suspension (target cells: 8 × 10^6^ cells/ml) at a ratio of 1:1. The resulting solution was incubated (30 min, 20 °C) with continuous and moderate shaking and the amount of released haemoglobin (Hb) was calculated after centrifugation, reading the absorbance of the supernatant at 541 nm.

The degree of hemolysis was determined using the equation: percent hemolysis = (measured release − spontaneous release)/(complete release − spontaneous release) × 100.

Previous hemocyte mortality in MS medium, under experimental conditions, was about 5%. Complete hemolysis (100% of red blood cells) was evaluated dissolving the erythrocyte suspension in distilled water.

Control erythrocyte suspensions were also prepared in the same medium and incubated as reaction mixtures: spontaneous haemoglobin release (hemolysis) never exceeded 5% of the total hemolysis.

### Statistical analysis

All the experiments were performed in triplicate. The value used was the mean of the three assays ± SD.

Analysis of variance (two-way ANOVA) on ranked data was applied to identify differences between all the different concentrations of the organometal considered significant for *p < 0.05, **p < 0.01 and ***p < 0.001.

Post hoc multiple comparison (Tukey test) was used where significant differences were detected in the ANOVA.

### Chemicals

Unless otherwise reported, all the used chemicals were from Sigma (USA).

## Results

### Mortality

**Fig. 1:**
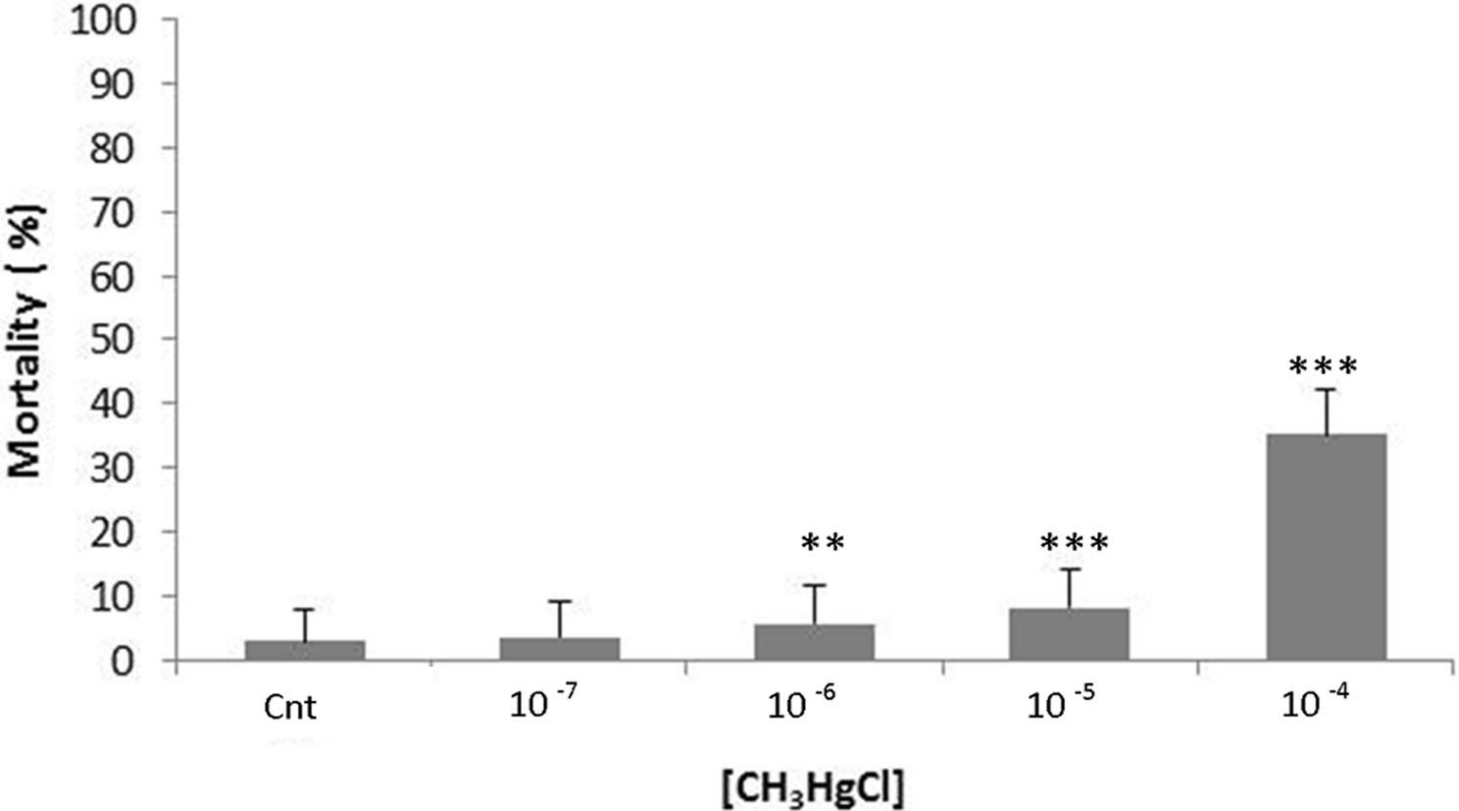
Effects of CH_3_HgCl on *M. galloprovincialis* hemocytes. The results are the mean of three culture replicates per treatment with corresponding standard deviation. Mortality by Trypan blue exclusion test was evaluated on cells incubated for 30 min in Marine solution (Cnt) and in the presence of different concentrations of CH_3_HgCl (10^–4^ M, 10^–5^ M, 10^–6^ M, 10^−7^ M). Treatment at concentration above 10^–5^ M of organic metal significantly reduces the vitality of the hemocytes. This indicates a statistically significant differences from the control group (one-way ANOVA and Tukey’s post hoc test, **p < 0.01 and ***p < 0.001)

One of the early steps before evaluating hemocyte activities is the evaluation of vitality. The mortality rate is determined by the Trypan blue exclusion test. The graph in Fig. 1 shows the mean ± SD of the mortality values, expressed as a percentage of the hemocytes considered as control (Cnt), after exposure to the xenobiotics (CH_3_HgCl 10^–4^ M, 10^–5^ M, 10^–6^ M, 10^−7^ M) for a period of 30 min.

An exposition at a concentration higher than 10^–4^ M of mercury resulted in a significant decrease in viability, which was lethal for the cells. As shown in the graph, a significant value for the concentrations of 10^–4^ M, 10^–5^ M and 10^–6^ M of MeHg has been found. The Trypan blue test results indicate that the treatment with a concentration higher than 10^–5^ M of the metal is able to reduce significantly the vitality of the hemocyte. Thus, all the following experiments were carried out considering the three sub-lethal concentrations of 10^−5^ M, 10^−6^ M and 10^–7^ after exposition for 20 min.

### Effect of methylmercury on hemocyte morphology

**Fig. 2:**
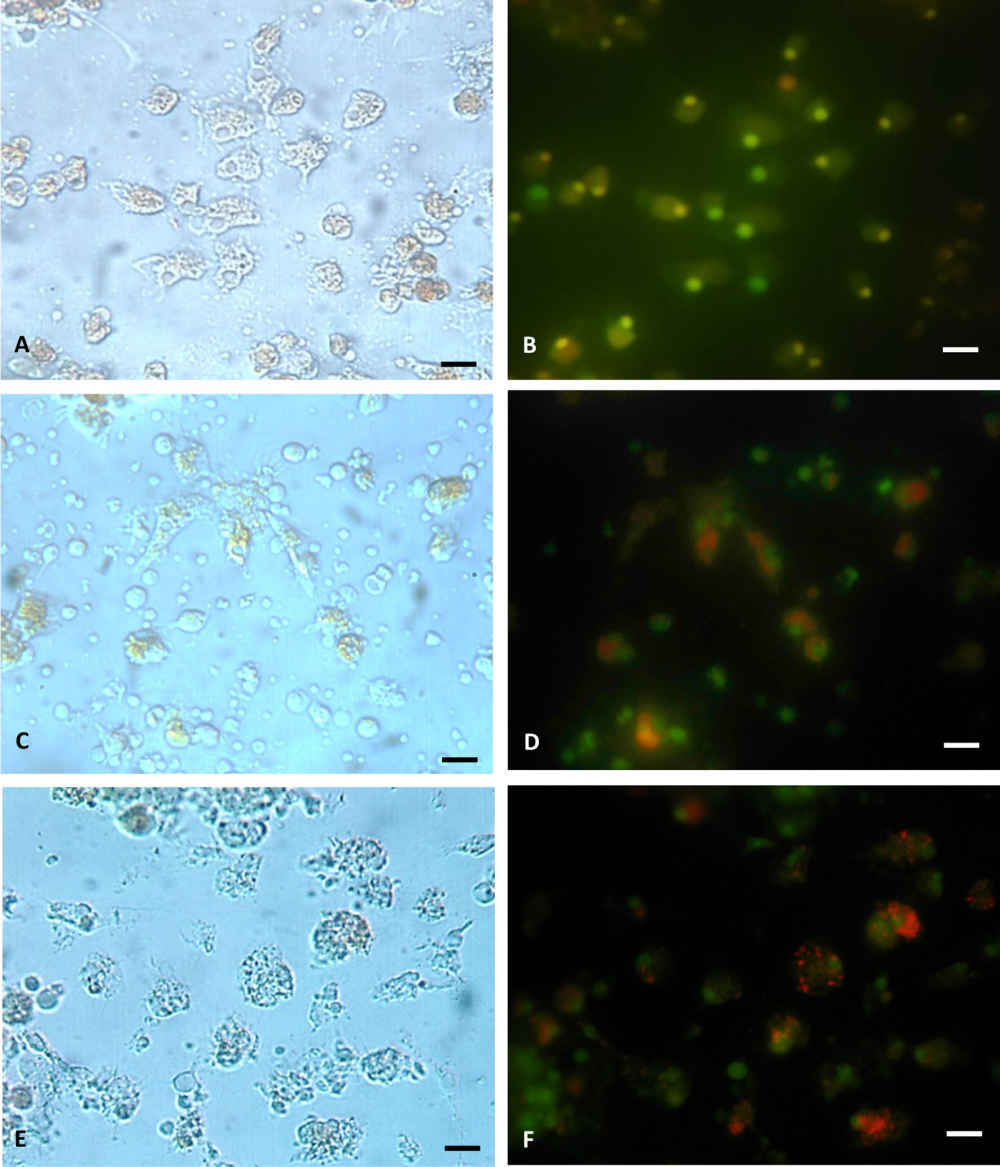
Representative images of hemocytes in marine solution (MS) after acridine orange (AO) staining. Observations of cells were carried out by light microscope with filter fluorescence in control condition (MS) (**a**, **b**). Hemocytes treated in MS with 2-deoxy-D-ribose and were stained with the vital dye AO (**c**, **d**) to detect apoptotic event. After incubation in 10^−5^ M MeHg, hemocytes appeared more rounded (**e**, **f**) but no apoptotic behaviors were found. Bar 10 µm

The pictures of the specimens viewed with the light microscope (Fig. 2a, c, f) and the fluorescence (Fig. 2b, d, f) are shown in Fig. 2. Almost all of the hemocytes stained by acridine orange (AO) maintained the motility and the ability to spread and adhere to the glass, showing a variable morphology (Fig. 2a, b). The positive control was realized by treating the hemocytes with the 2-deoxy-d-ribose and staining by AO (Fig. 2c, d).

**Fig. 3:**
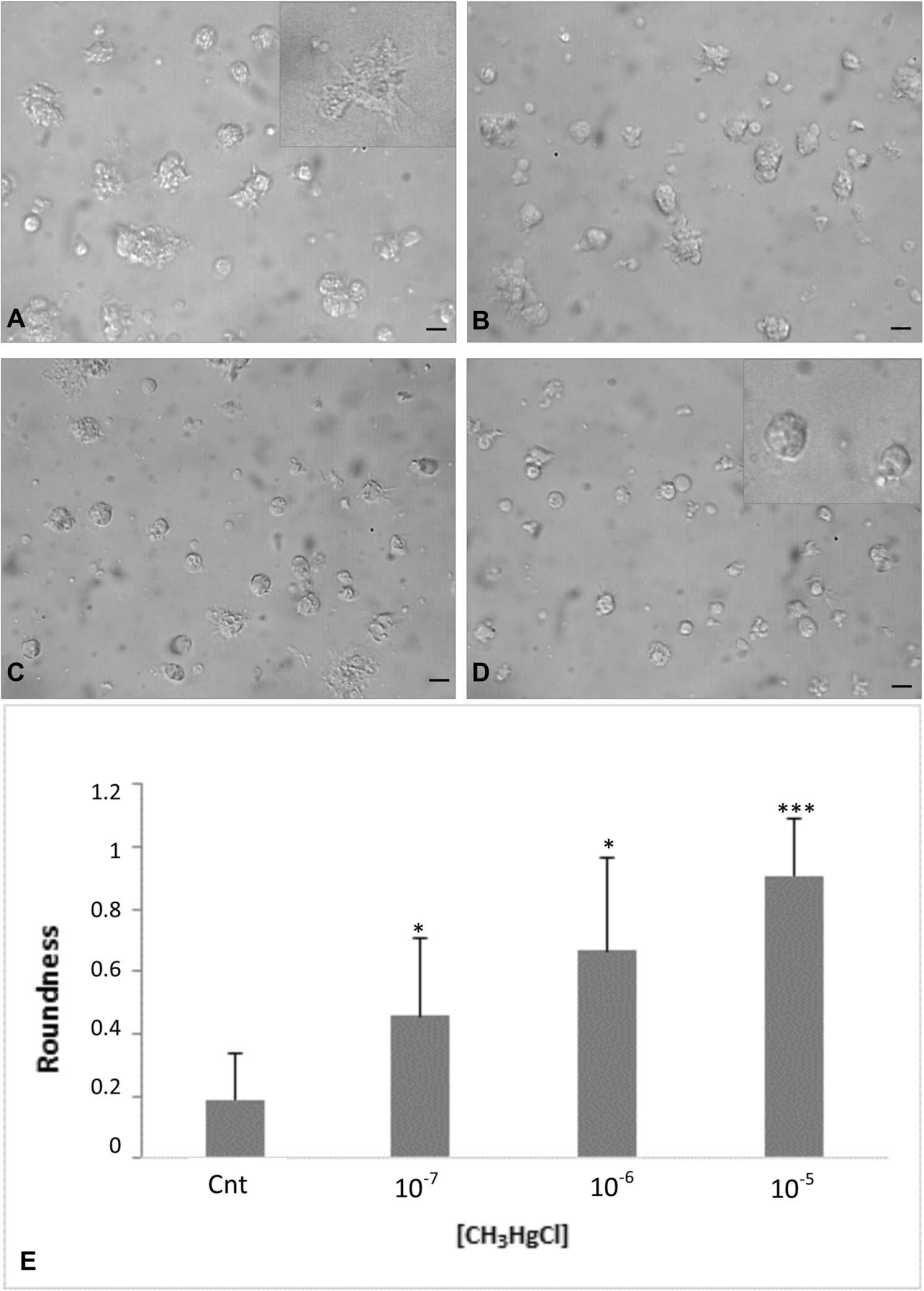
Change in morphology hemocytes *M. galloprovincialis* following exposure to methylmercury and quantification of roundness effect. Hemocytes were maintained for 20 min in MS in the absence of xenobiotic (**a**) and subsequently at various CH_3_HgCl concentration: 10^–7^ M (**b**), 10^–6^ M (**c**) and 10^–5^ M (**d**). Hemocytes in MS spread on the slide with elongated shape and pseudopods as is shown in magnification in **a**. After xenobiotic exposure at sublethal concentration, the cells undergo contraction and rounding (magnifications in **d**). Bar 10 µm. The results of the quantification of cell roundness, obtained from image processing by Image J software, are reported in **e**. The cells were incubated in MS and at the indicated MeHg concentration. Values are the mean of three replicates for treatment with the corresponding standard deviation bars. Asterisks indicate statistically significant differences between treatments (one-way ANOVA and Tukey’s post hoc test, **p < 0.01 and ***p < 0.001)

No apoptotic cells were observed after exposure at the lowest sub-lethal concentration, despite that the morphology was altered; in fact, the cells were rounder than the ones in the control resuspended in MS (Fig. 2e, f). In Fig. 3, the xenobiotic induced a modification in the shape of the hemocytes. On the contrary, in its absence, the cells were able to adhere to the substratum, showing variable and dynamic shapes (Fig. 3a). Thirty minutes later, on exposure to MeHg at various concentrations (10^–7^ M, 10^–6^ M and 10^–5^ M), the hemocytes changed shape proportionally with margins smooth relative to the controls (Fig. 3b–d, respectively).

After treatment with 10^–5^ M of MeHg, the number of cells appeared to have reduced (data not shown), and only round cells were observed (Fig. 3d).

Illustrations of the cell magnification of spreading in MS (Fig. 3a) and the contraction of the pseudopods and the round shape in the presence of xenobiotics at sublethal concentration (Fig. 3d) have been inserted.

The average values of the quantification of the cellular roundness are indicated in the graph in Fig. 3f. The control cells maintained in MS (Cnt) have the lowest roundness value (0.21). For the 10^–6^, 10^–5^ and 10^–4^ M methylmercury concentrations, the calculated roundness values proportionally increased (0.45 at 10^–7^ MeHg; 0.66 at 10^–6^ MeHg; 0.90 at 10^–5^ MeHg).

### Effect of methylmercury on phagocytosis

**Fig. 4:**
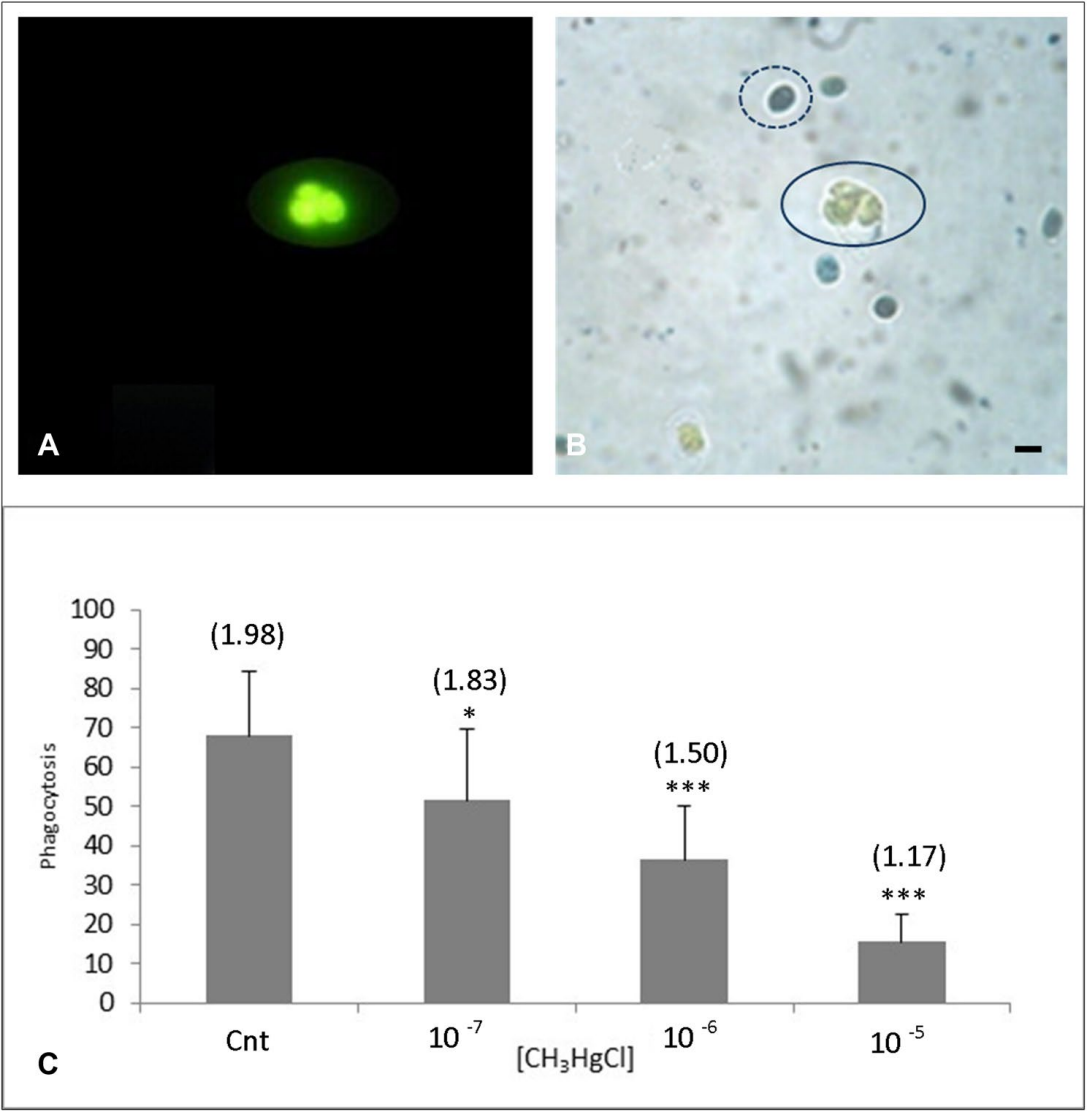
Phagocytosis of hemocytes of *M. galloprovincialis* towards yeast (*S. cerevisiae*). A: representative image of fluorescein-treated yeasts engulfed by phagocyte and observed by fluorescence microscope. B: same phagocyte cell detected in light field optical microscopy and indicated by the circle with a continuous line. The outline with a dotted line focused a non-internalized yeast within a phagocyte. Bar 10 µm. The graph reported the percentage of phagocytosis of *M. galloprovincialis* hemocytes to yeast under control conditions (MS) and after exposure to various MeHg concentrations. Each value is the result of the mean ± D.S. of three replicates. Asterisks indicate statistically significant differences between treatments (one-way ANOVA and Tukey’s post hoc test, **p < 0.01 and ***p < 0.001). Above each column of the graph, corresponding to the various concentrations of the xenobiotic, results of phagocytosis index (total number of yeasts/total number of phagocytes) are reported

In Fig. 4a, an illustration of the phagocytic capability of hemocytes, collected from *M. galloprovincialis*, towards yeast cells pre-stained by fluorescein under control conditions (MS) is reported. Looking through the fluorescent apparatus, the yeast engulfed by the phagocyte appeared to be fluorescent. An illustration of the phagocytes observed under the light microscope equipped with the Nomarski interference phase contrast is shown in Fig. 4b. Inside the circle with a continuous line is indicated a phagocytic cell. Indicated with a dotted line is a quenched uninternalized yeast. The parameters that permitted the highest phagocytic activity were a time period of 20 min and a temperature of 20 °C for the incubation. These parameters were considered for further experiments.

In the graph in Fig. 4c, data on the effect of phagocytes exposed in vitro to methylmercury are presented. Just after 20 min of incubation of the hemocytes in MeHg at 10^–5^ M, the yeast engulfment was much depleted.

Comparing with the control in MS, there was an evident decrease in and dose dependence of the phagocytic activity in the presence of MeHg at concentrations of 10^–7^ (*p* < 0.01) and 10^–6^ M (*p* < 0.001) (Fig. 4d). Lower values were observed with the contaminant at 10^–5^ M (*p* < 0.001).

The phagocytic index values are reported in Fig. 4c in brackets above the columns of the histogram and were obtained as the ratio of the total decrease in yeasts to the total decrease in phagocytes, with increasing MeHg concentrations used in the assay.

### Stability of the lysosomal membrane

On investigation of the stability of lysosomal membranes using the neutral red test, we saw that exposure to methylmercury resulted in a general decrease in the absorption of the NR solution.

**Fig. 5:**
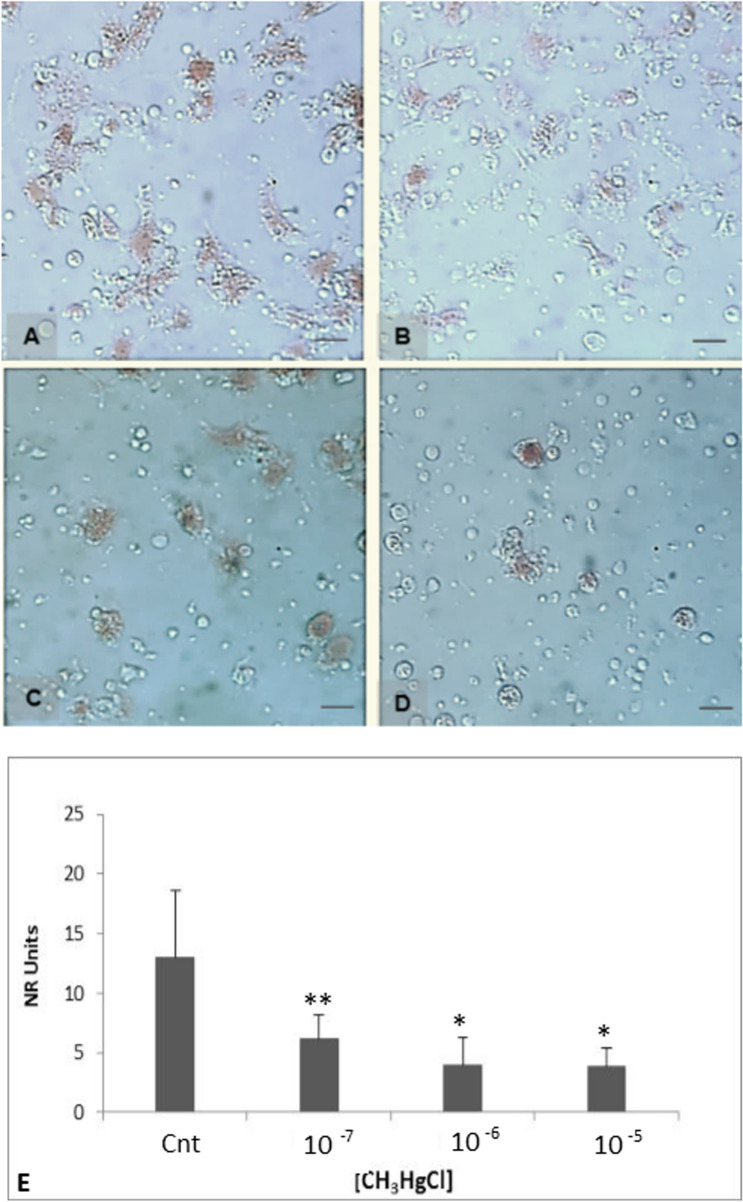
Trend of stability of the lysosomal membrane in the haemolymph samples of *M. galloprovincialis.* In the figure, mussel hemocytes show a variable uptake of neutral red in lysosomes. These organelles in healthy cells from individuals of M. galloprovincialis and incubated in MS take up and retained larger quantities of neutral red (**a**) than those from damaged cells post treatment in MeHg at 10–7 M (**b**), 10–6 M (**c**) and 10–5 M (**d**). The dye and the progress of dye uptake into the cells were visualized using a light microscope. Bar 10 µm. Lysosomal alterations are quantified by uptake of neutral red assay calculated on ODT/mg^−1^ ml^−1^ hemocyte protein and reported in **e**. Each value is the result of the mean ± D.S. of three replicates. Significant differences were calculated by Tukey’s post hoc test: *p < 0.05, **p < 0.01 and ***p < 0.001

In the same figure, the hemocytes with retaining of NR solution are shown. The intensity of the colour red is variable and related to the MeHg concentration. Under control conditions (Fig. 5a) and in the presence of the lowest concentration of MeHg (10^−7^ M) (Fig. 5b), the cells maintained their elongated shape and retained sufficient NR solution in their lysosomes. Despite that, the cells were treated with MeHg at 10^–6^ M and 10^–5^ M (Fig. 5c, d, respectively), the cells showed round shapes and the NR solution was not retained in the organelles, indicating a great destabilization of the integrity of the lysosomal membranes.

**Fig. 6:**
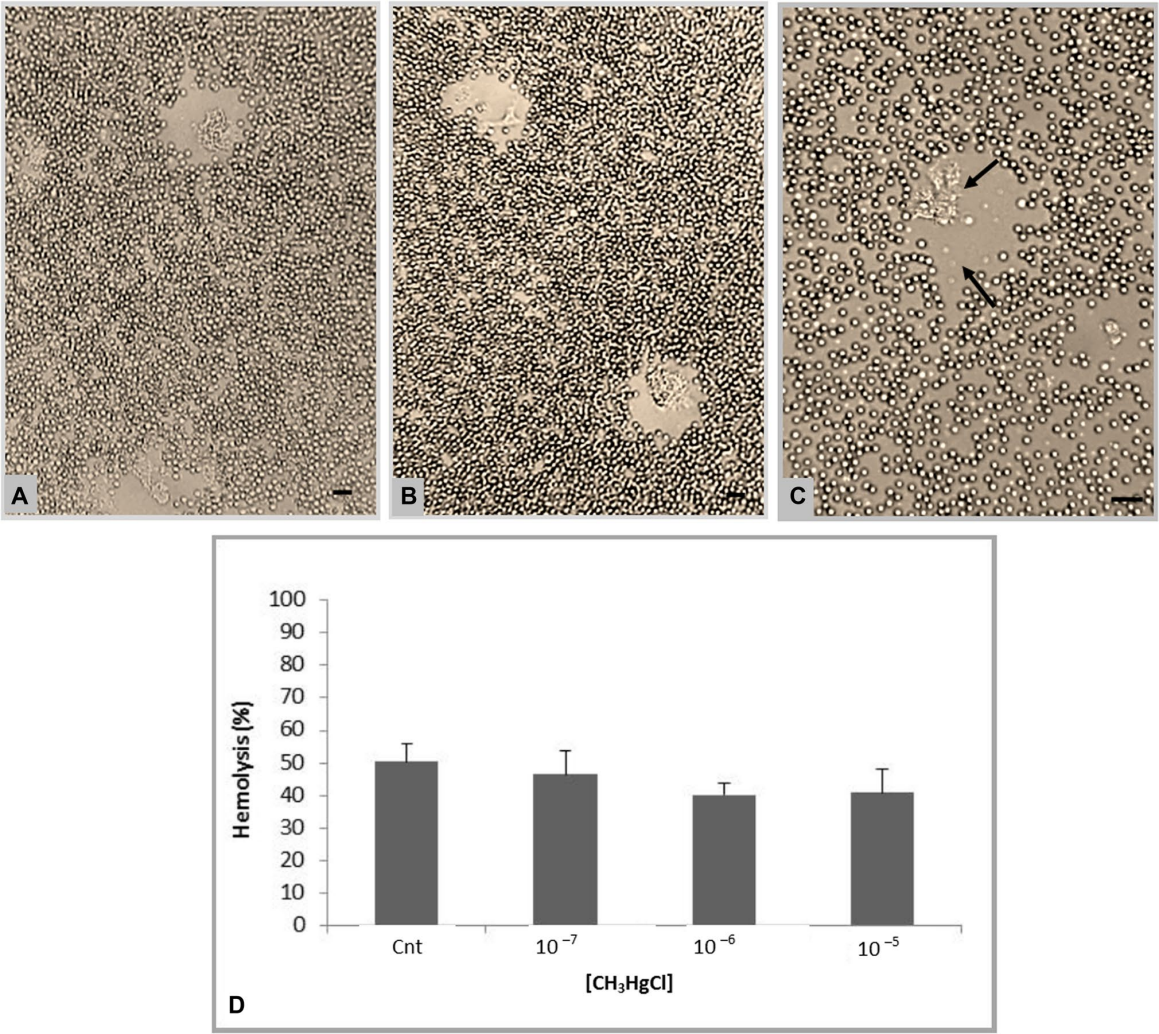
Plaques of lysis realized using *M. galloprovincialis* hemocytes as effector and pig erythrocytes as target cells. Images of lysis plaques toward erythrocytes (target 8 × 10^6^ cells) observed at the optical microscope (× 25) were carried out using hemocytes (effector, 1 × 10^6^ cells) pre-incubated in MS (**a**) and with 10^–5^ M MeHg concentration (**b**). Bar 10 µm. In **c**, the arrows indicate the hemocytes effector and the ghosts of lysed erythrocytes. Bar 20 µm. In **d**, the histogram showing mean ± D.S. of the cytotoxic activity values of *M. galloprovincialis* hemocytes to rabbit erythrocytes under control conditions and post treatment in MeHg at 10^–7^ M, 10^–6^ M and 10^–5^ M. There are no significant differences (*p* > 0.05)

Therefore, the organomethal treatment drastically reduced the retention of neutral red inside the cells as reported in graph in Fig. 5e. Additionally, a significance value of *p* < 0.05 was determined about the three MeHg concentration used for the assay (10^7^, 10^–6^ and 10^–5^ M.

### Plaques of lysis and cytotoxic activity of *M. galloprovincialis* hemocytes

Figure 6a shows the plaques of lysis visualized after the interaction between hemocytes and pig red blood cells (PiRBC) in a specific chamber meant for the experiment. The test was carried out in the presence of CH_3_HgCl at a sub-lethal concentration (10^–5^ M), and in this condition, the hemocytes expressed their cytotoxic capability to cause PiRBC lysis (Fig. 6b). In the magnification, shown in Fig. 6c, the arrow indicates the cell with a high probability of lysine release and responsible for the ghost erythrocytes

In the cytotoxic assays, the PiRBC hemolysis rate was evaluated under control conditions and in the presence of methylmercury at concentrations of 10^–7^ M, 10^–6^ M and 10^–5^ M. As can be clearly seen from Fig. 6d, the highest hemolysis value was individuated under control conditions and it was around 50%. From the data shown in the figure, it is clear that all the remaining values were comparable and that the presence of the metal did not have any significant effect on the cytotoxic activity towards the pig erythrocytes.

### ANOVA analysis

Table 1 shows the results of the univariate statistical analysis (ANOVA). In that analysis, the four cellular biomarkers of the mussel hemocytes were subjected to incubation at four distinct concentrations of MeHg.

**Table 1:** ANOVA analysis on immunological biomarkers

Biomarkers	Mortality		Phagocytosis		Uptake of NR		Hemocyte roundness		Cytotoxicity	
	*F*	*p*	*F*	*p*	*F*	*p*	*F*	*p*	*F*	*p*
Intercept	240.5	0.000	396.3	0.000	164.5	0.000	116.0	0.000	411.1	0.000
Treatment	42.12	0.000	28.88	0.000	20.04	0.000	7.42	0.000	0.21	**0.864**

Results of the univariate cellular assays (mortality, phagocytosis, hemocytes roundness and cytotoxicity) and changes in the lysosomal stability (neutral red uptake) on *M. galloprovincialis* hemocytes incubated with different concentrations (10^–5^; 10^–6^ and 10^–7^ M) of CH_3_HgCl. These analyses were carried out using the STATISTICA 10.0 software (StatSoft Inc. USA). *F* Fisher distribution. *p* test significance value. Only the *p* value of cytotoxicity biomarker, marked in bold (*p* = 0.864), is not significant according to statistic *p* test values. The differences resulted not significant for cytotoxicity (*p* > 0.05)

Particularly, mortality, phagocytosis, uptake of NR, hemocyte and cell roundness exhibited statistically high significant values (*p* < 0.01), while cytotoxicity was not significant (*p* < 0.5).

## Discussion

Heavy metals are often bioaccumulated in organisms at higher levels of the food chain, especially benthic animals (Wang 2002). There is need to develop strategies for assessing whether a particular environment is under stress or not. Therefore, measurement-based techniques biological effects are fundamental for any pollution-monitoring program (Viarengo et al. 2007).

The application of biomarkers in field conditions has been proposed by many authors to evaluate chronic responses in aquatic populations exposed to realistic environmental conditions.

Over the years, some marine bivalves, as mussels and clams, have been used extensively as biomonitors for metal pollution in marine coastal areas and for the screening to the characterization of pollution “hot spots” in environmental samples (Boening 1999; Farrington et al. 2016).

In vitro approaches are used to investigate the effects of chemicals responsible for pollution on the marine compartment. Particularly, toxic effects on immunity after exposure to pollutants have been studied by monitoring cellular immune parameters of sentinel marine organisms to validate biomarkers for systematic monitoring programs of coastal marine areas (Casas and Bacher 2006; Kim et al. 2008).

Inorganic mercury released from natural or anthropogenic sources becomes toxic in the environment when it enters a cycle involving bacteria and is converted to an organic form (methylmercury) (Muresan et al. 2008). It is highly toxic and its bioaccumulation in marine organisms is responsible for the mortality of aquatic species (Li et al. 2010; Rodrigues et al. 2013; Afshan et al. 2014; Bełdowska and Falkowska 2016).

Here, we evaluated the specific cellular responses to various concentrations of methylmercury in the Mediterranean bivalve representing a great economic food source (Boening 1999; Bresler et al. 1999; Calisi et al. 2007; Pérez-Camacho et al. 2014; Bellante et al. 2016; Beyer et al. 2017; Briant et al. 2017; Vaughn 2018).

The main purpose of using various concentrations of methylmercury was to determine the toxicological risk for organisms and also for human health, following the ingestion of contaminated edible species (Rocher et al. 2006; Li et al. 2010).

After treatment with 10^–5^ M MeHg, the lowest methylmercury concentrations that we used to examine the effect on immunocytes were not toxic, as indicated by the Trypan blue dead cell exclusion test, while viability was not affected by treatment, as demonstrated by the neutral red test. Generally speaking, 95% of the hemocytes maintained their main vital properties, while immune functions were affected by the xenobiotic compound. The cells incubated with various concentrations of MeHg diluted in artificial seawater showed a clear alteration of the cell surface and the spreading ability.

First, the roundness of the cells increased, while their density decreased in the field of the optical microscope in a dose-dependent fashion.

The analysis of the cellular roundness carried out by the image J processing software allowed detecting the presence of a few and short filopodia at the lowest concentration (MeHg, 10^–7^ M), while no filopodia were observed at the maximum concentration (MeHg, 10^–5^ M).

In a previous paper, it was demonstrated how MeHg was able to cause a change in the spreading capacity, in a dose-dependent manner (from 10^–7^ to 10^–4^ M), in the tunicate *Styela plicata* hemocytes (Cammarata et al. 2007; Parrinello et al. 2017). These effects suggest that Hg-induced alterations interact with cytoskeletal components. Particularly, Hg is able to inhibit tubulin polymerization and also induce microtubule disassembly (Pendergrass et al. 1997) modifying the SH groups of tubulins, decreasing cellular F-actin content (Sweet et al. 2006), and altering the phosphorylated and non-phosphorylated forms of cofilin, which regulate actin dynamics and facilitate actin filament turnover (Vendrell et al. 2010). Additionally, studies on snail hepatopancreas cells have demonstrated the impact of metals on the actin cytoskeleton (Manzl et al. 2004). In marine mussel, mercury alters the intracellular Ca^2+^ concentration (Marchi et al. 2004) that is a prominent regulator of the structure and dynamics of the cytoskeleton.

The regulation of cell volume, the cellular form, and the presence of pseudopodia are modulated by the action of the cytoskeleton (Hoffmann and Mills 2001). The cytoskeleton has a central role in the cell architecture; the effects of methylmercury on the cytoskeleton network could be evaluated as a cell shape parameter in exposed organisms.

Hemocyte-mediated phagocytosis of non-self-particles provide natural immunity in the bivalves (Iwanaga and Bok 2005; Rosales and Uribe-Querol 2017). One of the most consistent findings among potential targets of toxic heavy metals, especially mercury, is their effects on phagocytic cell function.

In invertebrate and vertebrate organisms, phagocytosis is well conserved and there is high sensitivity of this biological function to environmental xenobiotics in several animal species, emphasizing the usefulness of this approach in pollution monitoring programs (Wong et al. 1992; Voccia et al. 1994; Cooper et al. 1995; Dailianis 2011).

In this work, incubation of cells with MeHg reduced phagocytosis, accompanied by low changes in cell viability.

The possible effect on the cytoskeletal network seems to be confirmed by the morphological test optimized in this study in which the presence of methylmercury affected the morphology of the cells.

Lysosomal membrane stability and cytotoxicity were also determined in the control and test animals, to verify the applicability of hemocyte enlargement response in a biomarker panel to the bio-indicator organism as well as to a prognostic indicator for putative pathologies. Results showed variable staining intensity in a dose-dependent manner. With respect to the control, in the hemocytes subjected to high methylmercury concentration, the NR probe was less retained by the cell organelles, indicating an alteration of the integrity of the lysosomal membrane.

Destabilization of the lysosomal membrane has been demonstrated in response to a wide variety of stressors, such as metals, PAHs and persistent organic pollutants (POPs), and is considered a general biomarker of stress (Hwang et al. 2004; Hegseth et al. 2011) across a wide range of animals (Scott-Fordsmand and Weeks 2000; Regoli et al. 2006; Calisi et al. 2011). Thus, due to the consequences of the disturbance of cell functions, resulting in cell and tissue degeneration and the release of hydrolytic enzymes to the cytosol, the maintenance of lysosomal membrane integrity has been gaining special scientific attention from the immunological point of view.

Unlike the other markers, the in vitro cytotoxicity towards erythrocytes and the lysis formation assay on erythrocyte soil did not return any significant results. The presence of MeHg at the various concentrations did not seem to alter the ability of cells to release potential lytic molecules. Nothing is known yet about cytotoxicity towards other targets.

Cells capable of cytotoxic effects on erythrocytes have been characterized in other bivalves such as *Mytilus edulis* (Leippe and Renwrantz 1988) and *Cerastoderma edule* (Russell-Pinto et al. 1994), but there are no data on organometallic interaction.

From all results, we have shown that methylmercury is an active pollutant that causes cell death or in vitro modulation of hemocyte activity in a short period of time. In particular, the reported study shows experimental evidence that hemocyte morphology and phagocytic cells may be used as biomarkers of immunotoxicity in macrobenthic studies.

## Conclusion

The release of contaminants into the environment causes alterations in the ecosystem, especially in living organisms. Toxic metals have the propensity to pile up in benthic sessile organisms, which in turn may enter into the human metabolism owing to consumption, leading to grave health hazards.

The monitoring of ecosystem status, or biomonitoring, is largely based on the study of the ecotoxicological impact of xenobiotics through simple and reliable methods specifically adapted to the study of pollutant effects on living organisms at sublethal concentrations.

In this context, in vitro approaches were used in this study to understand the impact of methylmercury on *M. galloprovincialis* hemocytes. The mechanisms of cellular immunity of the mussel, as well as the structure of the hemocytes, appeared modulated by the effect of methylmercury, but not cytotoxicity.

Results confirmed that in vitro tests are, therefore, useful, sensitive and selective tools for the evaluation and monitoring of contaminants in marine environments.

The variations in hemocyte parameters could be considered potential biomarkers of the physiological status of the economic mollusk species. Moreover, the interactions between immunocompetence, disease susceptibility and pollutants in marine mollusks should be investigated, since animals with disturbed defence mechanisms due to pollutants may be more susceptible to infectious diseases.
